# Influenza virus infection during pregnancy: a review of risk factors, pathogenic mechanisms, and prevention strategies

**DOI:** 10.3389/fimmu.2026.1853575

**Published:** 2026-08-04

**Authors:** Huanhuan Fan, Dingyang Liao, Bingying Li, Zhejin Sheng, Bin Zhou, Limei Li

**Affiliations:** 1 Affiliated Hospital of Nanjing University of Chinese Medicine, Nanjing, China; 2 Research Center for Translational Medicine, Key Laboratory of Arrhythmias of the Ministry of Education of China, Shanghai East Hospital, Tongji University School of Medicine, Shanghai, China; 3 Nanjing University of Chinese Medicine, Nanjing, China; 4 School of Life Science and Technology, Tongji University, Shanghai, China; 5 Department of Vascular Surgery, Shanghai East Hospital, Tongji University School of Medicine, Shanghai, China

**Keywords:** influenza virus, maternal and child health, pregnancy, risk factors, vaccination

## Abstract

Influenza virus, as a highly mutable respiratory virus, has been persistently circulating in the human population and has become an important threat to global public health. Pregnant women are significantly more susceptible to influenza due to physiological changes in immune function, and their condition often becomes more severe after infection. Infection with influenza virus during pregnancy not only increases the severity of the disease and the risk of complications in pregnant women, but may also affect the neurological development and overall outcome of the fetus. This article systematically reviews the epidemiological characteristics, risk factors, and pathophysiological mechanisms underlying adverse maternal and infant health outcomes caused by influenza virus infection during pregnancy. It also explores in detail the safety and effectiveness of vaccination during pregnancy and evaluates the effectiveness of implementation comprehensive management strategies. By integrating the latest basic and clinical research results, this article aims to provide a literature reference for mechanistic research and the prevention and control of influenza virus infection during pregnancy, as well as scientific basis for clinical decision-making and optimization of public health strategies, promote effective control and management of influenza during pregnancy, and ensure the health and safety of mothers and infants.

## 1 Introduction

As a globally consequential infectious pathogen, influenza virus infection not only causes acute respiratory diseases but may also lead to severe complications, resulting in millions of cases and significant mortality annually, thereby posing a serious threat to public health and safety (1, 2). Although several vaccines and antiviral drugs are currently available for the prevention and treatment of influenza, the virus’ high variability and complex transmission mechanisms impede eradication, resulting in recurrent outbreaks and even global pandemics (3, 4). Pregnant women face a significantly higher risk of influenza infection during flu seasons compared to nonpregnant women and are more prone to severe complications, a phenomenon widely supported by clinical observations and epidemiological evidence (5, 6).

Influenza virus infection presents a prolonged course in pregnant women, with some requiring hospitalization and even intensive care unit (ICU) admission (6). According to US Influenza Hospitalization Surveillance Network data between 2010 and 2019, approximately 27.9% of hospitalized influenza patients 15–44 years of age were pregnant women (7). Pregnant women infected with influenza face significantly higher risks of severe illness, such as higher ICU admission rates and mechanical ventilation requirements compared to nonpregnant individuals.

Influenza virus infection during pregnancy poses not only maternal health risks but also potentially impacts fetal and neonatal health. Multiple studies have shown that influenza infection during pregnancy significantly increases the risk of preterm birth and low birth weight, and may even threaten fetal life in severe cases (8, 9). Systematic reviews and meta-analyses confirm that pregnant women infected with influenza A and B viruses face a markedly elevated risk of preterm birth (8). Additionally, influenza infection may also affect fetal nervous system development and increase the risk of infections and illnesses in the neonatal period (10–12).

The current global prevalence of influenza virus and its impact on pregnant women and fetuses have garnered widespread attention. Ongoing fundamental and clinical research has deepened understanding and advanced prevention and treatment strategies, such as vaccination and antiviral therapy. However, insufficient vaccine coverage, misconceptions, and incomplete elucidation of pregnancy-specific immune mechanisms remain key areas for future research and clinical practice. This article aims to systematically summarize the epidemiological characteristics, susceptibility factors, pathological mechanisms, treatment strategies, and current preventive measures of influenza virus infection during pregnancy. It seeks to provide literature support for enhancing pregnant women’s awareness of the potential hazards of influenza virus infection and promote the development of more scientifically sound prevention and management strategies.

## 2 Epidemiological characteristics of influenza virus infection during pregnancy

### 2.1 Classification and epidemiological characteristics of influenza viruses

The influenza virus belongs to the Orthomyxoviridae family and is a single-stranded, negative-sense, segmented RNA virus. Based on the viral nucleoprotein and matrix protein composition, it can be classified into four types: A, B, C, and D (13). Types A, B, and C can infect humans, while type D rarely infects humans (**Table 1**).

**Table 1:** Classification and epidemiological characteristics of influenza viruses.

Type	Infected species	Virus transmission	Subtypes	Variant	The risk of infection among pregnant women	Reference
IAV	Humans, other mammals, and poultry	Cross-species transmission between animals and humans	Multiple subtypes. Among the multiple subtypes, the most common are H1N1 and H3N2.^*^	High variability	The predominant circulating subtypes of influenza viruses mainly include H1N1 and H3N2 influenza A viruses; the risk of severe illness in pregnant women infected with the H1N1 virus is significantly higher than that associated with other subtypes. Infection with the H3N2 subtype is associated with a higher incidence of complications in pregnant women, but its mortality rate is lower compared to the H1N1 subtype.	(1, 2, 6, 9, 13, 14)
IBV	Mainly infecting humans and seals	Transmission occurs mainly through interpersonal contact via droplets, leading to regional seasonal epidemics.^#^	Two branches, Victoria and Yamagata. Alternating epidemic characteristics	The mutation rate is lower than that of type A.	Pregnant women with influenza B virus typically present with relatively mild clinical symptoms.	(13, 15–17)
ICV	Mainly infecting humans and pigs	Mostly sporadic infections	No subtype classification was identified.	The antigenicity of the virus has remained remarkably stable since its initial isolation in 1947.	More than 75% of children show evidence of infection, whereas adult cases often present asymptomatically or with a mild cough.	(13, 18)
IDV	Primarily infects cattle and pigs, rarely infects humans. It is not included in the human seasonal influenza surveillance system.					(13)

^*^Pandemic influenza primarily results from infection with influenza A viruses.

^#^Seasonal influenza is mainly caused by the IAV subtypes H1N1 and H3N2, and influenza B virus.

IAV, influenza A virus.

IBV, influenza B virus.

ICV, influenza C virus.

IDV, influenza D virus.

Global influenza pandemics, such as the 1918 Spanish flu (H1N1) and the 2009 swine flu (H1N1), have primarily been caused by the influenza A virus (IAV) (14). IAV can infect humans, other mammals, and poultry, among which poultry are natural hosts (19, 20). Cross-species transmission between animals and humans may lead to virus recombination and the formation of new strains (19). IAV has multiple subtypes, classified based on different combinations of hemagglutinin and neuraminidase on the surface of the virus envelope (14, 21). Common subtypes include H1N1 and H3N2.

Relative to IAV, influenza B virus has a more limited transmission range and host range, mainly infecting humans and seals. Furthermore, the influenza B virus mutation rate is slower than that of IAV, usually leading to regional seasonal epidemics and milder symptoms. Influenza B virus is divided into two branches, Victoria and Yamagata, which exhibit alternating epidemic characteristics (15). Victoria and Yamagata viruses alternate as the dominant branch, and their epidemic frequencies differ in different regions (15–17). Additionally, the age distribution of influenza B virus patients differs between the two branches, with Yamagata more likely to infect older patients, and Victoria more likely to infect school-aged children 5–15 years of age (16, 17).

The pathogenicity of influenza C virus is relatively weak, similar to the common cold, and it rarely causes serious diseases or epidemics (18). Infections are generally sporadic with relatively small impacts on public health. Serological studies have shown that influenza C virus infections generally occur in children 7–10 years of age, but its epidemiological burden may be underestimated due to difficulties in cell culture testing (18).

Overall, IAV is characterized by diverse subtypes and rapid mutation. The influenza B virus exhibits two main branches of alternating prevalence, namely the seasonal influenza virus. The influenza C virus infects more children, but its epidemiological characteristics are not yet fully understood.

### 2.2 Cross-species transmission of the influenza virus

The influenza A virus is the influenza virus possessing the highest capacity for cross-species transmission. Its host range encompasses a diverse array of species, including humans, birds, pigs, cats, and ferrets. Therefore, it is a prototypical zoonotic pathogen (13, 14). In contrast, the primary hosts of influenza B and C viruses are humans. Although they are occasionally detected in animals such as pigs and seals, their host range is considerably narrower than that of the influenza A virus (13). Based on their primary host, cross-species influenza viruses can be classified into avian influenza virus, swine influenza virus, and human influenza virus (14).

#### 2.2.1 Avian influenza virus

The avian influenza virus plays a pivotal role within the influenza A virus family (19). The avian influenza virus is capable of infecting not only over 100 bird species but also crossing species barriers to infect various mammals, including humans (22, 23). The flowchart illustrates the cross-species transmission of the avian influenza virus: (A) “Sources of the virus”, which primarily includes wild bird reservoirs and environmental reservoirs (19). Wild birds, especially waterfowl of the order Anseriformes, are the main natural reservoirs of avian influenza virus. The virus replicates in their intestines without causing disease; this mode of replication distinguishes it from other types of influenza viruses. Subsequently, it is excreted via feces, thereby contaminating water sources and the broader environment. Environmental reservoirs include contaminated live poultry markets, water sources for breeding farms, and similar settings, where viruses can persist and serve as a source of transmission. (B) The “intermediate links of transmission” encompass various pathways such as direct contact, indirect contact, aerosols, and vectors. Direct contact is the primary mode of transmission among poultry, while indirect contact is mediated by contaminated media such as feed, drinking water, and utensils. Aerosol transmission also constitutes a significant route under specific conditions, such as high-density aquaculture environments. (C) Vulnerable hosts include poultry, wild birds, mammals, and humans. Poultry is a key link in virus amplification and transmission, while mammals such as pigs, minks, and seals can serve as “ mixing vessel “ to promote reassortment of different virus subtypes (24, 25). As the definitive host, humans are primarily infected through contact with infected poultry or environments contaminated by them. (D) Infection outbreaks and the emergence of new strains. The virus continues to circulate in susceptible hosts, giving rise to new genotypes or subtypes through reassortment, thereby increasing the risk of a pandemic (26).

#### 2.2.2 Swine influenza virus

Pigs are widely regarded as the primary “mixing vessel” for influenza viruses, as their respiratory epithelial cells co-express the avian-specific alpha-2,3 sialic acid receptor and the human-specific alpha-2,6 sialic acid receptor (19, 27). This unique receptor profile facilitates co-infection with avian, human, and swine influenza viruses (19, 27). This provides an ideal environment for viruses reassortment from diverse origins and the subsequent emergence of new strains capable of cross-species transmission. Two main routes exist for cross-species transmission of the swine influenza virus: the pig-human transmission pathway and the pig-poultry-human triple transmission network. The transmission of swine influenza virus from pigs to humans is a critical step in cross-species transmission, and this transmission typically initiates through direct contact between humans and infected pigs or contaminated environments (28, 29). The dynamic process of the triple transmission network involving pigs, poultry, and humans includes viral circulation within the pig population, the introduction of avian influenza virus into the pig population via feces or contaminated water, and the entry of human influenza virus into this population via farm workers (24, 30). Pigs act as “mixing vessels,” thereby facilitating the genetic reassortment of swine, avian, and human influenza viruses (25, 27). If the new strain acquires the ability to bind to human receptors, it can be transmitted from pigs to humans through close contact during activities such as feeding and slaughtering, or through aerosol transmission; some strains can spill back into poultry populations or cause limited human-to-human transmission, thereby establishing a sustained transmission cycle (24, 30).

#### 2.2.3 Human influenza virus

Human influenza viruses have fully adapted to the human host, but seasonal epidemic strains (such as H1N1 and H3N2) can still transmit back to animals, establishing a cross-species transmission cycle, a phenomenon known as “reverse zoonosis” (31). For example, in the Brazilian swine population, human seasonal H1N1, H1N2, and H3N2 viruses have been introduced independently at least 8 times since the 1980s, establishing distinct genetic lineages that continue to circulate within the swine population (32).

The transmission cycle of the human influenza virus comprises the following steps: Step 1: Viral infection of the host. The main target cells of the human influenza virus are epithelial cells in the upper respiratory tract (including the nasal cavity, pharynx, and trachea), which support efficient viral replication. Step 2: The host expels newly generated human influenza viruses through coughing, sneezing, and exhalation. Step 3: The virus persists and remains viable in the environment until it eventually infects a new host (33, 34).

The host range of the influenza virus is continuously expanding, and its high genetic variability is increasing the risk of cross-species transmission from avian and other animal reservoirs to humans. To reduce the risk of infection, particularly for pregnant women, direct contact with live poultry, poultry carcasses, and their droppings should be avoided. Poultry meat and eggs should be thoroughly cooked before consumption. Individuals with a history of contact with poultry who develop flu-like symptoms should seek immediate medical attention and inform their healthcare provider of the potential exposure.

### 2.3 Epidemic characteristics and the risk of infection in pregnant women

The prevalence of influenza virus during pregnancy has significant seasonal and regional differences, and these variations directly affect the risk of pregnant women contracting influenza virus. Worldwide, the influenza virus typically spreads once a year, mainly during the winter season. In the northern hemisphere, it is typically prevalent from October to April of the following year, while in the southern hemisphere, it is more prevalent from April to October (5, 35). Influenza activity in tropical regions shows year-round fluctuations, with less pronounced seasonality than in temperate regions (35). Therefore, the infection rate among pregnant women significantly increases during their region’s peak influenza season.

Additional factors also affect the risk of influenza virus infection in pregnant women. Climate factors such as temperature and humidity regulate the spread of influenza virus and therefore affect the risk of infection in pregnant women. Low temperature and low humidity conditions are conducive to the survival and transmission of viruses, thereby promoting the spread of influenza and increasing the susceptibility of pregnant women to the invasion of influenza viruses (36, 37). During the COVID-19 pandemic, global public health interventions to combat the spread of the SARS-CoV-2 virus also disrupted the spread of influenza virus, with some areas experiencing abnormal phenomena such as delayed flu season or even summer outbreaks (38).

Overall, climate, geographical location, and public health interventions can all influence global and regional influenza epidemic characteristics. Pregnant women are at high risk for influenza complications, and their infection rates and prognosis are closely related to the influenza season and virus prevalence.

### 2.4 Different rates of infection with influenza virus subtypes and variants for pregnant women

Among the influenza virus subtypes, numerous epidemiological studies have confirmed that pregnant women are primarily susceptible to the IAV subtypes H1N1 and H3N2, and influenza B virus lineages, such as Victoria and Yamagata (5). Moreover, the IAV subtypes H1N1 and H3N2 continuously undergo genetic mutations during the epidemic, especially in the hemagglutinin and neuraminidase genes, thereby affecting viral transmission and pathogenicity (21). For example, the H3 subtype virus exhibits strong adaptability and can maintain high transmission capacity under high temperature and humidity conditions (39).

#### 2.4.1 H1N1 subtype

IAV subtype H1N1 infections cause significantly higher rates of severe illness and mortality in pregnant women than H3N2 and influenza B viruses. A US study from 2010 to 2019 showed that the risk of severe illness in pregnant women infected with H1N1 virus was significantly higher than other subtypes, with an adjusted risk ratio of 1.9 (7). This finding is also supported by research from other countries: A study from Brazil revealed that pregnancy is an independent risk factor for severe H1N1 infection (40). Moreover, pregnant women infected with H1N1 experience rapid disease progression and have a poor prognosis (40). In summary, among pregnant women, H1N1 subtype infection leads to significantly increased disease severity, hospitalization and mortality rates, and risks of maternal and infant complications.

#### 2.4.2 H3N2 subtype

IAV subtype H3N2 infection exhibits a higher incidence of complications in pregnant women, but a lower mortality rate compared to the H1N1 subtype. Multiple epidemiological studies have shown that H3N2 is the most common IAV subtype, and is especially dominant during the winter and spring seasons. For example, in a retrospective analysis from 2021 to 2024 of influenza-like cases in the Chongqing High-Tech Zone, H3N2 was the predominant IAV subtype, accounting for a significant proportion of influenza virus infections (41). Similarly, in the school influenza outbreaks from 2020 to 2023 in Jiangsu Province, China, H3N2 showed stronger adaptability, often co-circulating with other subtypes such as H1N1 and leading to larger scale outbreaks (42).

#### 2.4.3 Influenza B subtype

Pregnant women infected with influenza B virus are often asymptomatic or present with mild symptoms (31). The influenza B virus is mainly divided into two lineages, Victoria and Yamagata, which exhibit varying prevalence based on geography and season (43). For example, seasonal influenza virus testing results from 2013 to 2019 in Yichang City, Hubei Province, China, showed that Yamagata was prevalent from 2013 to 2016, while B/Victoria was prevalent from 2016 to 2019 (44). Influenza outbreak data from 2018 to 2024 in Shenzhen, China, indicated that B/Victoria caused a relatively high frequency of outbreaks, but usually at a small scale (45). Although influenza B virus usually results in asymptomatic or mild symptoms in infected populations, its frequent latent infections increase the risk of virus transmission and the difficulty of prevention and control.

IAV subtype H1N1 infection often leads to more severe clinical symptoms and higher hospitalization rates in pregnant women, while IAV H3N2 and influenza B virus infections cause relatively mild clinical manifestations. Notably, the clinical symptoms of influenza B virus in particular are relatively mild in pregnant women, often presenting with mild or no obvious symptoms, and therefore easily overlooked. This phenomenon leads to an underestimation of the actual infection rate of influenza B virus, which in turn affects the development of public health intervention strategies.

### 2.5 Seasonal influenza and pandemic influenza

Seasonal influenza is mainly caused by the IAV subtypes H1N1 and H3N2, and influenza B virus. These viruses routinely experience antigenic drift, where the surface antigens of the virus undergo small variations year by year (46, 47). These variations decrease antigen recognition by antibodies formed from previous infections or vaccines, which increases the viral ability to evade the immune system and decreases immune protection, thereby triggering periodic outbreaks of seasonal influenza (46, 47). This antigenic drift requires influenza vaccines to be updated annually to match prevalent virus strains and maintain vaccine effectiveness.

In contrast, pandemic influenza is usually caused by new strains of influenza virus, which are often produced through antigenic shift, namely, the recombination of different viral gene segments or the emergence of new subtypes, which can lead to a general lack of immunity in the population and trigger large-scale outbreaks (21, 48). For example, the 2009 H1N1 pandemic influenza virus is a novel recombinant virus with high infectivity and cross-species transmission ability, leading to a global influenza pandemic (49).

Both seasonal and pandemic influenza viruses spread primarily through droplet and contact transmission routes, infecting respiratory epithelial cells and causing disease (46). However, due to the lack of a population immune barrier, the pandemic influenza virus spreads faster with a wider range of infections than seasonal influenza viruses, leading to rapid global spread (48). Furthermore, pandemic influenza viruses typically have stronger cell receptor affinity and higher replication ability, leading to a higher host viral load and therefore greatly increased infectivity and transmission efficiency (50).

In summary, seasonal influenza viruses maintain low-level variation through antigen drift, leading to local or regional epidemics. Pandemic influenza viruses arise from new virus subtypes generated through antigenic shift, leading to a global outbreak with more harmful effects, due to the lack of immune protection in the population against the new virus.

### 2.6 Differences in the risk of infection among pregnant women at different stages of pregnancy

The risk of infection among pregnant women varies through the different stages of pregnancy. Infection risk has been shown to be higher in the late stages of pregnancy (especially after 24 weeks), and increases further near delivery (7). The hospitalization and mortality rates of pregnant women infected with influenza virus in late pregnancy are significantly higher than those of nonpregnant women and early-stage pregnant women (7). During the 2009 H1N1 influenza pandemic, pregnant women were more likely to have pulmonary complications in the second and third trimesters, leading to an increase in incidence rate and mortality (51). Furthermore, in pregnant women infected with influenza virus, the perinatal mortality rate increased five-fold and the premature birth rate increased four-fold (52).

The types of maternal and infant risks caused by influenza virus vary at different gestational stages. Influenza virus infection during early pregnancy increases the risk of miscarriage and abnormal fetal development, while infection during late pregnancy is more likely to cause severe maternal complications and premature birth (8, 53). Other studies have shown that influenza virus infection during early pregnancy is associated with an increased risk of fetal brain development abnormalities and neurological disorders (54–56). Therefore, to strengthen protection and early intervention for pregnant women, influenza prevention strategies should be differentiated for the different stages of pregnancy, especially during the peak influenza season.

Influenza virus infection during pregnancy has distinct epidemiological characteristics, including high incidence rate, seasonal fluctuations, and regional differences. Additionally, disease severity varies with different virus subtypes. Seasonal climate factors and peak epidemic periods significantly affect the risk of influenza virus infection in pregnant women, and the severity and outcome of influenza infection vary at different stages of pregnancy.

## 3 Risk factors for susceptibility to influenza virus infection during pregnancy

**Figure 1:**
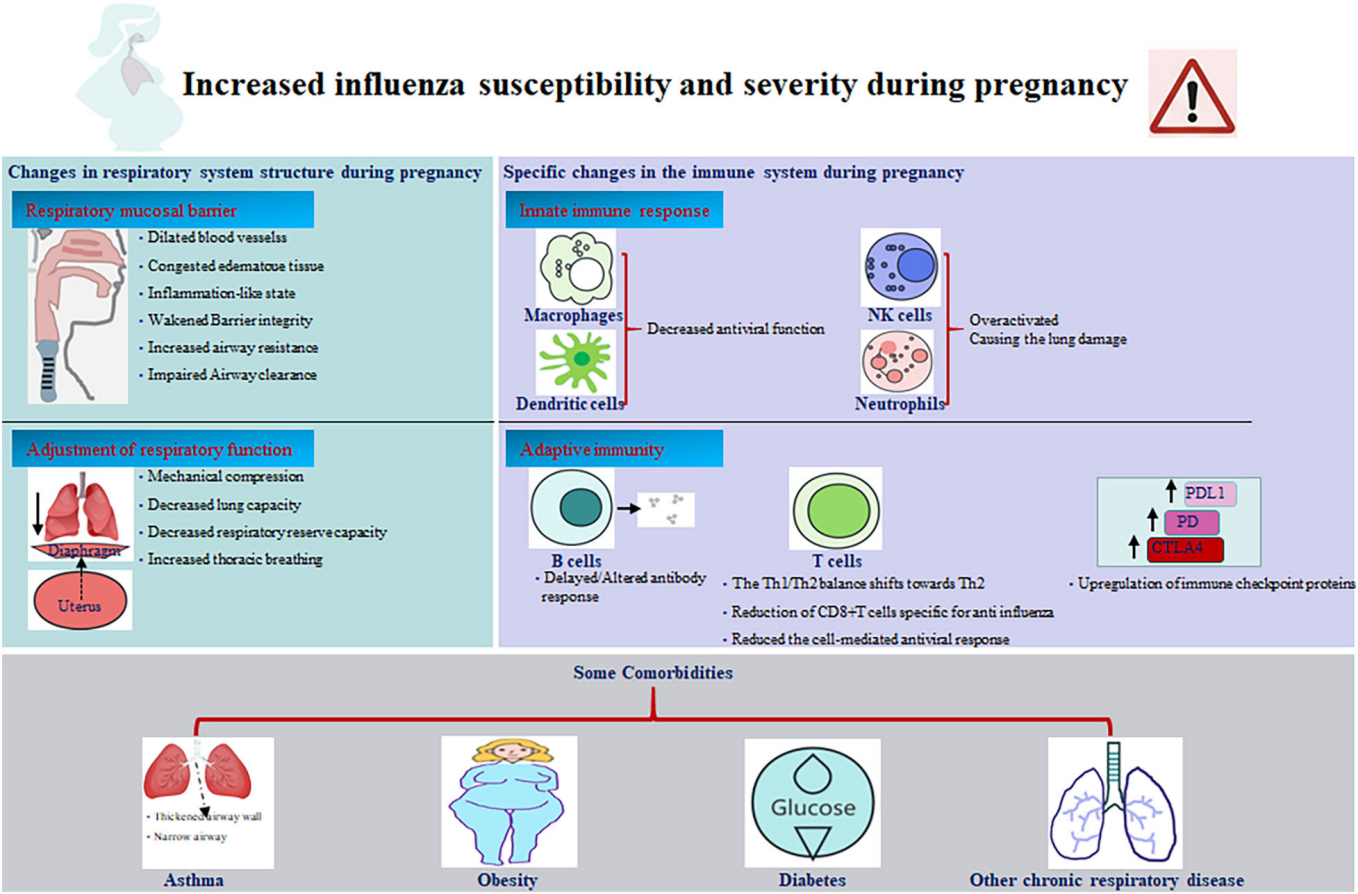
Risk factors for susceptibility to influenza virus infection during pregnancy. Certain physiological changes unique to pregnancy, namely, changes in respiratory system structure and immune system regulation, are key factors that heighten the susceptibility of pregnant women to influenza. Common chronic underlying diseases, including asthma, obesity, pre-pregnancy diabetes, and chronic respiratory diseases, are important risk factors during pregnancy that affect the risk of influenza virus infection and exacerbation of illness.

Certain physiological changes unique to pregnancy, namely, changes in respiratory system structure and immune system regulation, are key factors that heighten the susceptibility of pregnant women to influenza. Additional factors, such as age and diabetes status of pregnant women, also affect the infection rate of influenza during pregnancy (**Figure 1**).

### 3.1 Changes in respiratory system structure and susceptibility to influenza

#### 3.1.1 Changes in respiratory mucosal barrier

During pregnancy, the respiratory mucosal barrier undergoes significant physiological changes that may affect the integrity of the mucosal barrier. The increased estrogen levels during pregnancy can cause dilation, congestion, and tissue edema of nasal mucosal blood vessels, while stimulating increased glandular secretion (57). A controlled study confirmed that women in late pregnancy had poorer nasal patency compared to the nonpregnant control group, and further examination showed that pregnancy-related nasal mucosal swelling led to impaired upper respiratory airflow (57). This mucosal edema and congestion are not limited to the nasal cavity, but also involves the entire upper respiratory tract, constituting changes in the first physical barrier of respiratory defense during pregnancy.

The inflammatory response status of nasal and tracheal mucosa also changes during pregnancy. Although pregnancy-related physiological mucosal vasodilation and edema are themselves an inflammatory-like state, external stimuli may trigger stronger inflammatory responses (57, 58). Two experiments on mice have shown that exposure to environmental pollutants, such as ozone, during pregnancy—especially in late pregnancy (gestational day 13–18)—significantly exacerbates the ovalbumin sensitization-induced asthma phenotype in offspring, manifesting as pulmonary inflammatory infiltration, mucus secretion, and deterioration of the Th2-type immune response (59).

During pregnancy, the respiratory mucosal barrier exhibits increased blood flow and mucus secretion, as well as weakened barrier integrity due to hormonal and environmental factors. These changes in the respiratory mucosal barrier in pregnant women determine the overall susceptibility of the respiratory tract to influenza virus and the pathological progression after infection.

#### 3.1.2 Mechanical adjustment of respiratory function

During pregnancy, the uterus gradually enlarges and mechanically compresses the diaphragm, resulting in a significant decrease in lung capacity and respiratory reserve capacity (60, 61). Restricted diaphragm activity hinders lung expansion, reduces lung compliance, and ultimately affects the overall function of the respiratory system. Mechanical compression also causes changes in chest movement patterns, prompting pregnant women to adopt chest breathing compensation, thereby increasing the burden of respiratory work. Pulmonary function tests show that pregnant women have varying degrees of decline in lung function indicators such as lung capacity, functional residual capacity, and expiratory flow rate, especially in the late stages of pregnancy (60). Increased airway resistance also commonly occurs during pregnancy. This increase in airway resistance is partly due to edema and increased airway mucosa secretions (61). Increased airway resistance limits the airway clearance function, allowing viruses and other pathogens to more easily remain in the respiratory tract, exacerbating the risk of infection (61).

Animal model studies have also shown that mechanical pressure can induce remodeling of respiratory structure and function, such as morphological changes in airway epithelial cells and weakened respiratory muscle function (62). During pregnancy, the weakening of lung oxygenation function and respiratory muscle strength further reduces resistance to infection and increases the incidence of serious complications such as respiratory failure (60).

Therefore, the mechanical adjustment of the respiratory system during pregnancy, especially the compression of the diaphragm and increased airway resistance, significantly weakens respiratory function and defense capabilities, creating favorable conditions for the occurrence of respiratory infections such as influenza virus.

### 3.2 Specific changes in the immune system during pregnancy and susceptibility to influenza

The human immune system combats viruses in two stages, the innate immune response and adaptive immune response, which eliminate viruses through the synergistic effects of physical barriers, immune cells, and antibodies. The immune system during pregnancy exhibits unique adaptive adjustments that result in maternal immune status and immune tolerance.

#### 3.2.1 Innate immune response

The nasal epithelial cells are the main gateway for virus entry and the primary site of innate immune defense in the host. Influenza virus entry into host cells requires the binding of its surface protein hemagglutinin to the sialic acid receptor on the host cell surface, followed by endocytosis into the host cell; therefore, sialic acid receptors mediate the entry of influenza virus into nasal epithelial cells (63). Research has shown that sialic acid receptor expression is significantly upregulated and accompanied by enhanced interferon (IFN)-α release in pregnant rat, indicating that the innate immune response in the respiratory tract is regulated during pregnancy (64). This finding may partially explain the increased sensitivity of pregnant women to IAV infection and the specificity of the immune response.

During pregnancy, complex changes occur in the function of local natural immune cells that resists influenza virus invasion: First, progesterone modulates the activity of macrophages and dendritic cells, suppresses their production of pro-inflammatory cytokines, and impairs their ability to clear viruses (65, 66). Second, many studies have confirmed that NK cell activity also increases during pregnancy (67). In pregnant women, the increased NK cell activity in severe cases of influenza reflects the strong activation of the body’s natural immune system to combat the virus under intense viral stimulation. However, in severe cases, this activation often loses control and develops into overactivation and triggers tissue damage (68). This finding has also been validated in mice (69–72). Third, research has shown that pregnancy can lead to an increase in neutrophils in peripheral blood (73). Pathogens can induce neutrophil extracellular traps, high levels of which can exacerbate acute lung injury in influenza (74). In addition, the expression level of immature neutrophil markers increases linearly during pregnancy, and this immune state increases susceptibility to certain infections (75).

The expression of sialic acid receptors, which mediate the entry of influenza virus into cells during pregnancy, is significantly upregulated in the respiratory tract. However, innate immune defense is intricately regulated by hormones, resulting in weakened macrophage and dendritic cell function, and thereby leading to decreased antiviral ability. Due to their overactivation, NK cells and neutrophils cause immune hypersensitivity and damage to the lungs, increasing the risk and severity of influenza virus infection in pregnant women.

#### 3.2.2 Adaptive immunity

The humoral immune response is weakened in pregnant women, which decreases viral clearance and increases susceptibility to influenza virus. This impaired influenza virus clearance is related to pregnancy-induced changes in multiple humoral immune response mechanisms: First, antibody production is delayed. Studies have shown that expression of antibody production-related genes is downregulated in pregnant women infected with influenza virus, which hinders antibody maturation and affects viral clearance (69). Another study found that estrogen levels significantly increase during pregnancy, thereby delaying the production of virus-specific humoral immune responses and leading to delayed antibody production against H5N1 virus in pregnant women, further increasing the risk of infection (76). This elevated estrogen concentration was also shown to reduce B cell-driven lymphangiogenesis in pregnant mice (77). Second, the abundance of different antibody types changes. Gordon et al. found that pregnant women who were severely infected with pH1N1 during pregnancy showed a decrease in serum IgG2 levels, indicating that IgG subclasses play a role in the outcome of pH1N1 infection (78). In Shenyang, China, in 2009, H1N1-infected pregnant woman produced different proportions of anti-H1N1 IgG1, IgG2, IgG3, and IgG4 antibody subtypes compared to H1N1-infected nonpregnant women in the same hospital (79). Third, antibody structure and function are altered. For example, changes in antibody glycosylation levels lead to impaired effector function, especially for antibodies targeting the H1 subtype, which may affect the ability to protect pregnant women against influenza virus infection (80–82). Fourth, memory B cells change during pregnancy. The number of CD27+ memory B cells are reduced during pregnancy, especially during the second and third trimester, which may lead to a weakened humoral immune response and increased susceptibility to influenza virus in pregnant women (68). However, studies have also reported an increase in memory B cells after pregnancy, with a decrease in transitional B cells and anergic naïve subsets (83). Therefore, the changes in memory B cells during pregnancy are not a single quantitative change, but rather a redistribution between subgroups and a reshaping of functional states. Some subgroups may decrease, while others may increase or activate.

The cellular immune function of pregnant women is also partially suppressed, mainly mediated by T cell-mediated immune responses. First, the proportion of T cell subsets undergoes significant changes during pregnancy; specifically, the Th1/Th2 balance shifts towards Th2, which reduces the cell-mediated antiviral response (68). Th1 cells are mainly responsible for secreting pro-inflammatory cytokines such as IFN-γ, which play a role in clearing viruses. Therefore, this decreased IFN-γ secretion during pregnancy directly affects influenza virus clearance efficiency. Elevated progesterone during pregnancy is a primary driver of shifting the immune system towards Th2 and therefore promotes the establishment of immune tolerance. Second, influenza-specific cytotoxic CD8+ T cells are reduced. Pazos et al. reported that during pregnancy, the number of influenza-specific CD8+ T cells is significantly decreased in the lungs and lymph nodes, and total and influenza-specific CD8+ T cell migration in the lungs is delayed, causing insufficient activation of CD8+ T cells and delayed virus clearance in mice (84). This finding has also been validated in pregnant women. Savic et al. reported that after pregnant women were infected with influenza virus, compared with asymptomatic cases, the frequency of IFN γ+CD8+T cells with restricted influenza A virus epitopes was generally reduced in symptomatic cases (85). Sridhar et al. reported a similar finding: individuals reporting mild influenza symptoms exhibited higher frequencies of pre-existing T cells specific to influenza A CD8 epitopes (86).

Research has shown that the upregulation of immune checkpoint proteins (such as PDL1, PD1, and CTLA4) during pregnancy leads to the formation of an immunosuppressive state, thereby increasing the susceptibility of pregnant women to influenza virus infection and exacerbating disease severity (66, 87). For example, in a pregnant mouse model, administering anti-PDL1 antibodies effectively suppressed immune checkpoint proteins, enhanced antiviral immune responses, alleviated lung inflammation, reduced viral load, and improved lung function, indicating that pregnancy-induced immune suppression is a key factor in the severity of influenza virus infection (66).

During pregnancy, B cell function is inhibited, and the Th2-mediated immune response and upregulation of immune checkpoint proteins is enhanced. Although this immune state is beneficial for fetal tolerance, it reduces the ability to clear influenza virus. In summary, immune tolerance during pregnancy is one of the fundamental physiological mechanisms underlying the susceptibility of pregnant women to influenza infection.

### 3.3 Comorbidities and susceptibility to influenza

The risk of infection is significantly increased for specific groups of pregnant women, such as those with underlying diseases. Common chronic underlying diseases, including asthma, obesity, pre-pregnancy diabetes, and chronic respiratory diseases, are important risk factors during pregnancy that affect the risk of influenza virus infection and exacerbation of illness.

#### 3.3.1 Asthma

Asthma increases the risk of contracting influenza virus and disease severity in pregnant women. Specifically, asthma can cause respiratory inflammation and increased airway hyperresponsiveness in pregnant women, which exacerbates the severity of influenza virus infection (9). A prospective study showed that 71% of asthmatic pregnant women contracted colds, compared to 46% of nonasthmatic pregnant women. Furthermore, these asthmatic pregnant women were significantly more likely to experience multiple colds with higher symptom severity during pregnancy relative to nonasthmatic pregnant women (33% and 16%, respectively) (88). In another study, 60% of asthmatic pregnant women with respiratory viral infections experienced decreased asthma control and increased rates of preeclampsia. Influenza infection exacerbates and increases the severity of multiple asthma phenotypes (88). During the 2009 swine flu pandemic, pregnant women, especially those with underlying lung diseases such as asthma, often exhibited febrile diseases that developed into secondary pneumonia, leading to an increase in hospitalization rates (89, 90). Influenza infection exacerbates and increases the severity of asthma phenotypes in pregnant women.

Asthma impairs the IFN response, which may contribute to the increased susceptibility of pregnant women to influenza. Compared with healthy nonpregnant women, the IFN-λ response in pregnant asthma patients was significantly reduced; furthermore, during an acute asthma attack, IFN-αand IFN-λ were both decreased (91). Another study has found that nonallergic, granulocyte-deficient asthma patients show impaired production of type I IFNs. Additionally, mice with neutropenic asthma showed increased disease severity and influenza virus mutations after influenza infection, compared with the control group. Moreover, wild-type mice infected with influenza virus isolated from asthmatic hosts developed more severe disease (92). These data indicate that asthma patients are not only more susceptible to influenza infection in general but may also be more susceptible to severe influenza virus subtypes specifically.

Asthma can lead to decreased antiviral ability and increased inflammatory response in pregnant women. In pregnant women infected with human IAV H1N1pdm09, the asthmatic patients exhibited a weakened antiviral response in peripheral blood mononuclear cells (PBMCs) and primary nasal epithelial cells compared to the nonasthmatic group (93, 94). The PBMCs of pregnant women with gestational asthma also exhibit reduced antiviral and immune regulation functions, and increased inflammation (91). Vanders et al. found that IAV infection in early pregnancy exacerbated inflammatory asthma phenotypes and reduced antiviral responses in an asthma mouse model (95). In pregnant women with allergic airway disease, IAV infection is associated with impaired antiviral and antibacterial responses, increased lung inflammation, excessive mucus secretion, and airway hyperresponsiveness. In summary, asthma is associated with increased susceptibility to influenza virus infection and disease severity during pregnancy.

#### 3.3.2 Obesity

Obesity increases the risk of contracting the influenza virus. Results from a cross-sectional study of 1040 Japanese adults showed a significant correlation between higher visceral fat area and influenza infection rate (96). Interestingly, in pregnant women, obesity increases susceptibility to the H1N1pdm subtype specifically. Maier et al. recruited 335 IAV patients and 1506 family contacts and found that obesity was associated with increased susceptibility in adults to IAV/H1N1pdm, but not H3N2; furthermore, this association was found to increase with age, since obesity was not found to be associated with susceptibility to either H1N1pdm or H3N2 in children 5–17 years of age (97). Surprisingly, the association between obesity and H1N1pdm outcomes was shown to be stronger in women than in men. A cohort study of 194 pregnant women infected with pH1N1 influenza virus found that obesity during pregnancy increased the risk of serious consequences, including ICU admission, from pH1N1 infection (98).

The obesity rate during pregnancy continues to rise, and obese pregnant women have chronic low-grade inflammation in their bodies, accompanied by elevated levels of pro-inflammatory cytokines and adipokines (including TNF-α, IL-6, IL-1β, and leptin), impaired immune cell function, and abnormal response to infection. In a diet-induced obese ferret model, obesity was shown to cause significant changes in the lung microenvironment, thereby increasing the risk of influenza virus infection and allowing the virus to spread more easily to the lower respiratory tract, causing more severe disease symptoms (99). In summary, obesity, as an important component of metabolic syndrome, can exacerbate the inflammatory response during infection, leading to increased lung and systemic inflammation, and increasing the risk of respiratory failure and cardiovascular complications.

#### 3.3.3 Other risk factors

Other chronic diseases such as diabetes and chronic respiratory diseases also increase the risk of influenza infection in pregnant women. Pregnant women with diabetes are prone to infection due to poor blood sugar control, impaired immune function, decreased virus clearance after infection, and increased likelihood to develop a virus–bacteria coinfection, exacerbating their condition (100). Patients with chronic respiratory diseases already have impaired basic lung function, and respiratory symptoms worsen even further when invaded by influenza virus, thereby increasing the likelihood for hospitalization and mechanical ventilation (101).

In summary, due to anatomical and physiological changes in the respiratory system and unique immune system regulation during pregnancy, pregnant women are significantly more susceptible to influenza virus infection, with stronger disease severity, especially in the late stages of pregnancy. Risk factors for influenza virus infection during pregnancy also include chronic underlying diseases. Therefore, clinical prevention and control should comprehensively evaluate individual risks and develop individualized prevention and control strategies.

## 4 Maternal and infant health risks from influenza virus infection during pregnancy

**Figure 2:**
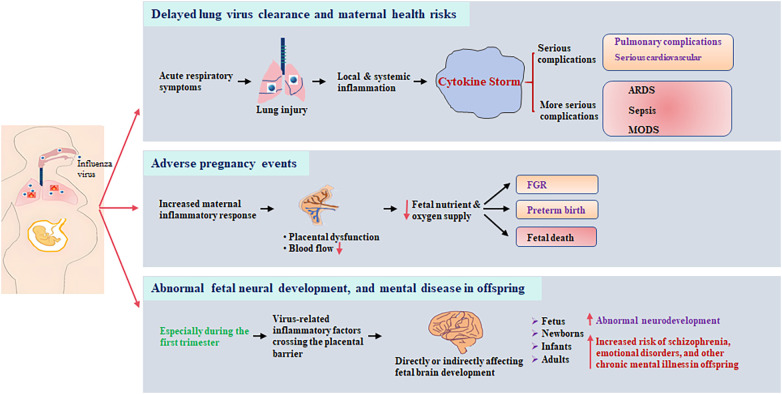
Maternal and infant health risks from influenza virus infection during pregnancy. Influenza virus infection not only poses significant direct health risks to pregnant women, such as acute respiratory symptoms, severe pulmonary disease, and serious cardiovascular complications, but they may also experience more serious complications, such as acute respiratory distress syndrome, sepsis, and multiple organ dysfunction, which can even be life-threatening in severe cases. It adversely affects pregnancy outcomes, significantly increasing the risk of adverse pregnancy events such as premature birth, fetal growth restriction (FGR), and fetal death. These adverse consequences not only threaten the short-term health of newborns, but may also lead to long-term neurodevelopmental abnormalities and chronic mental illness.

Influenza virus infection not only directly poses significant health risks to pregnant women, but also strongly affects pregnancy outcomes, significantly increasing the risk of adverse pregnancy events such as premature birth, fetal growth restriction (FGR), and fetal death. These adverse outcomes not only threaten the short-term safety of newborns but may also lead to long-term neurodevelopmental abnormalities and chronic mental illness (7–12). Research has confirmed that the transmission and pathogenesis of influenza virus during pregnancy are more complex than in nonpregnant populations (**Figure 2**).

### 4.1 Delayed lung virus clearance and maternal health risks

In addition to the acute respiratory manifestations of influenza virus infection, pregnant women may also develop more serious complications such as acute respiratory distress syndrome, sepsis, and multiple organ dysfunction, which can even be life-threatening in severe cases (5, 7). Severe lung disease is the most frequently reported outcome associated with the increased incidence rate of influenza infection among pregnant women, which indicates that lung involvement is an important factor that determines disease severity (7, 102).

Mouse studies showed deteriorated lung pathology and a higher viral load in infected pregnant mice relative to the nonpregnant infected control group, indicating that uncontrolled viral replication can lead to these pathologies (103, 104). These findings are consistent with the dampened antiviral immune response seen in pregnant women. As discussed in Section 3.2, the immune system of pregnant women gradually becomes suppressed to support fetal growth; however, this suppression also increases their susceptibility to influenza virus infection and reduces their ability to clear the virus after infection. The slow clearance of respiratory viruses in pregnant women triggers highly active innate and adaptive immune responses, leading to persistent local inflammation in the lungs and prolonged systemic inflammation, promoting inflammatory storms and ultimately causing serious cardiovascular and pulmonary complications (103, 105).

In summary, the immune response in the lungs during pregnancy exhibits delayed activation and overactivation. The activation of immune cells and the release of inflammatory factors in the lungs are delayed and excessive, leading to exacerbation of lung tissue inflammation and increased risk of pathological damage.

### 4.2 Embryonic dysfunction, premature birth, fetal growth restriction, and fetal death

Influenza virus infection during pregnancy significantly affects pregnancy outcomes, particularly preterm birth and FGR, conditions that can lead to fetal death in severe cases. A retrospective cohort study found that influenza infection during pregnancy, especially in the third trimester, significantly increases the risk of low birth weight and premature birth (106).

Numerous studies have shown that influenza virus cannot directly infect the placenta, indicating that influenza virus does not directly affect the fetus (107). However, the maternal inflammatory response induced by influenza virus infection can lead to placental dysfunction and reduced placental blood flow, thereby affecting the nutrition and oxygen supply of the fetus and increasing the risk of premature birth and FGR. Animal model studies have shown that influenza virus infection in pregnant mice can cause transient but severe placental cell death and loss of trophoblast cells, leading to placental tissue damage (108, 109). Moreover, the simultaneous infiltration of inflammatory cells in the placenta has significant effects, leading to an increase in pro-inflammatory cytokines, thereby decreasing progesterone levels, inducing placental dysfunction, and delaying fetal development (108, 109). A pregnant mouse model showed that IAV infection triggers a systemic “vascular storm”, in which numerous inflammatory cells infiltrate the peripheral vascular endothelial layer and secrete inflammatory mediators, leading to maternal vascular damage and dysfunction, especially to uterine placental circulation, thereby limiting placental blood perfusion and reducing nutrient delivery to the fetus (73, 110). This vascular inflammation not only causes placental hypoplasia but can also lead to intrauterine growth restriction and fetal hypoxia, causing premature birth and FGR in pregnant women.

In short, influenza virus infection during pregnancy induces the maternal inflammatory response, especially the “storm” of vascular inflammation caused by the activation of vascular endothelial cells, which damages maternal vascular function, reduces placental blood flow and function, causes insufficient fetal nutrition supply, and significantly increases the risk of premature birth, FGR, and even fetal death.

### 4.3 Maternal immune activation, abnormal fetal neural development, and mental disease in offspring

Epidemiological studies have shown that exposure to viral infections, including IAV, during pregnancy, especially during the first trimester when almost all organ systems are formed, significantly increases the incidence of neurodevelopmental disorders, schizophrenia, and mood disorders in offspring (111–113).

Although no evidence of direct viral infection in the fetal brain has been detected, influenza virus infection in pregnant women can cause virus-related inflammatory factors to cross the placental barrier, thereby directly or indirectly affecting fetal development, with a particularly profound impact on nervous system development. The maternal immune activation (MIA) model suggests that viral infection can activate T helper 17 cells in the maternal gut, producing the pro-inflammatory cytokine interleukin-17, which crosses the placental barrier and affects fetal brain development, leading to structural damage such as neocortex abnormalities (114). Additionally, microglia and marginal macrophages in the fetal brain are considered potential mediators of MIA-induced cortical abnormalities. These immune cells exhibit dose-dependent and time-dependent changes during the infection process, such as stable numbers of microglia but increased phagocytic activity (CD68 expression), indicating their involvement in inflammatory responses and brain tissue remodeling, further affecting neural development (115). Brain imaging studies have shown that immune responses triggered by viral infections during pregnancy lead to impaired development of brain regions and structures (116). A macaque study further confirms that influenza virus infection during pregnancy can cause abnormal changes in the white matter area of fetal astrocytes, which manifest as granular cytoplasmic inclusions accompanied by activation and reactive changes in microglia (117).

Inflammatory-mediated cytokines may not only lead to fetal brain structural abnormalities but may also affect the brain structure of newborns, infants, and even adults, leading to schizophrenia and emotional disorders in offspring (114). Injecting influenza vaccine antigen into pregnant Balb/c mice at E6 or E16 reduced the exploratory behavior of their offspring in the open arms of the Elevated Plus Maze. Further research suggests that MIA caused by influenza infection during pregnancy leads to behavioral deficiencies in mice (118). Other studies have shown that H1N1pdm09 infection in pregnant mice can lead to a decrease in specific areas of neurogenesis and an increase in glial response in 14-day-old offspring. The increased reactivity of astrocytes in the CA1 and dentate gyrus regions highlights the duration of neuroinflammatory effects. These results emphasize the role of the maternal influenza-induced immune response in shaping hippocampal development (119). After being infected with influenza during pregnancy, magnetic resonance imaging analysis showed that the offspring of rhesus monkeys exposed to influenza during pregnancy had significantly reduced gray matter in the cerebral cortex. The bilateral cingulate and parietal regions exhibited the greatest reduction in gray matter; additionally, the parietal region also showed significantly decreased white matter (120). The above results suggest that the inflammatory environment caused by influenza infection during pregnancy has a sustained negative impact on the construction of neural networks in offspring.

The neurological abnormalities in offspring caused by influenza virus infection during pregnancy not only stem from temporary neuropathological changes in the fetus due to direct maternal immune response, but may also trigger subtle long-term brain damage.

In summary, due to physiological changes in immune function, pregnant women may experience excessive immune responses and persistent inflammation after being infected with influenza virus. The inflammatory response caused by influenza virus infection during pregnancy has multilevel complexity, including the elevation of systemic pro-inflammatory cytokines and the involvement of pregnancy-specific immune regulatory mechanisms, leading to persistent and intractable inflammation, and exacerbating maternal and fetal tissue damage. This series of mechanisms not only explains the high risk of influenza during pregnancy but also provides a theoretical basis and potential targets for future immune regulation and anti-inflammatory treatment against influenza virus infection in pregnant women.

## 5 Prevention and treatment strategies for influenza virus infection during pregnancy

Vaccination and antiviral therapy are currently the primary prevention and treatment strategies for influenza virus infection during pregnancy.

### 5.1 The critical importance of influenza vaccination during pregnancy

#### 5.1.1 Benefits of vaccines

Vaccination against influenza in pregnant women can reduce the incidence of influenza virus infection and its serious complications, thereby protecting maternal and infant health. For example, a prospective study conducted in Greece from 2018 to 2019 showed that the laboratory-confirmed influenza infection rates were lower in pregnant women who received the quadrivalent inactivated influenza vaccine (2.1%) compared to unvaccinated pregnant women (7.5%). Therefore, vaccination reduced the risk of infection by approximately 72% (121).

Vaccination for pregnant women not only protects the mother but also protects infants under six months of age by transporting antibodies through the placenta, thereby reducing their risk of influenza-related hospitalization. A retrospective study in Spain estimated that influenza vaccination during pregnancy can prevent 61% of flu-related hospitalizations in infants under six months of age (122). During the high incidence period of influenza activity, maternal influenza vaccination was observed to have a protective effect on low birth weight, and a marginally protective effect on births with young gestational age (123).

Therefore, after receiving the influenza vaccine, pregnant women not only have a significantly reduced risk of self-infection but can also provide protection for newborns from the antibodies transported through the placenta. Pregnant women should therefore receive inactivated influenza vaccines at any stage of pregnancy to reduce the risk of influenza and its complications.

#### 5.1.2 Differences in maternal and neonatal antibody responses induced by different vaccines

Different vaccine types induce significant differences in the immune response in pregnant women, which in turn affects the protective ability toward both the mother and the newborn. Live, attenuated influenza vaccine and recombinant hemagglutinin vaccines can induce stronger and more persistent immune responses in pregnant women, as evidenced by higher levels of specific antibody production and more effective immune protection (124). Conversely, the traditional trivalent inactivated vaccine has a less robust protective effect, based on the induction of antibody subtypes and shorter protection duration (124).

#### 5.1.3 Vaccine safety

Vaccine safety is the core consideration in pregnancy vaccination decisions. Multiple studies have shown that maternal influenza vaccination has not been associated with spontaneous abortion, chorioamnionitis, pregnancy-induced hypertension, preeclampsia, pregnancy-induced diabetes, or preterm delivery (125, 126). There is no significant correlation between influenza vaccination during pregnancy and premature birth, stillbirth, small for gestational age infants, congenital defects, or serious maternal nonobstetric adverse events (127, 128). Multiple large-scale cohort studies have also shown no increase in adverse pregnancy outcomes, whether during the first vaccination of a singleton pregnancy or during two consecutive pregnancies (127, 129). The safety of influenza vaccines during pregnancy has been extensively studied, and studies overwhelmingly indicate that vaccination does not significantly increase the risk of adverse pregnancy outcomes, providing a scientific basis for the use of multiple vaccines during pregnancy. However, due to the complex regulation of the immune system during pregnancy, the use of live vaccines still requires caution, and most clinical guidelines recommend recombinant proteins and inactivated vaccines as the first choice.

#### 5.1.4 Current status of influenza vaccination, vaccine hesitancy and proposed response strategies

Globally, despite the significant protective effect of influenza vaccination for mothers and infants, influenza vaccination rates among pregnant women remain low. Significant variations exist across countries, regions, and healthcare systems, with vaccination rates ranging from the single digits to more than 70% (130–134). Data from the U.S. Centers for Disease Control and Prevention indicate that approximately 61.2% of pregnant women received the seasonal influenza vaccine during the 2019–2020 influenza season (130). Despite an increase in this proportion compared with prior years, it remains insufficient to encompass the entire spectrum of high-risk populations (130). A survey conducted in New South Wales, Australia, found that the self-reported influenza vaccination rate among pregnant women was approximately 54% (131). Although the technical guidelines for influenza vaccines in China explicitly recommend that pregnant women are eligible to receive inactivated influenza vaccines at any stage of pregnancy, the vaccination rate remains low because the vaccines are primarily an out-of-pocket expense, compounded by limited public awareness (132). In Australia, vaccination willingness differs among social and cultural groups, with lower vaccination rates among ethnic minorities and pregnant women with lower education levels (133). A study in Florida, USA, found that because the state’s Medicaid program does not cover the cost of influenza vaccination during pregnancy, the vaccination rate among pregnant women covered by this program is significantly lower than that of the privately insured population (134). In a cohort of 316 women, 70.4% of those with private insurance and 35.6% of Medicaid beneficiaries received the influenza vaccine during pregnancy (134). These data collectively depict a global challenge: although the benefits of influenza vaccination during pregnancy have been widely proven, achieving high and equitable vaccination coverage remains a significant challenge.

The global vaccination rate is generally below 50%, and vaccine hesitancy is the primary barrier (135). Vaccine hesitancy is a complex, context-dependent, and time-varying process, rather than a simple binary choice between ‘yes’ and ‘no’. Vaccine hesitancy is particularly pronounced during pregnancy. The key drivers of influenza vaccine hesitancy in this population include: (A) At the individual level, the primary obstacles to vaccination among pregnant women are safety concerns, including concerns about vaccine ingredients, potential adverse effects on fetal development, and uncertainty about vaccine effectiveness (136, 137). Furthermore, insufficient awareness of disease risks among pregnant women is also a significant factor; many women underestimate the severity of influenza’s impact on both themselves and their fetuses, thereby reducing their willingness to receive vaccinations (138, 139). (B) Sociocultural factors, such as negative information from family, friends, or social media, also significantly exacerbate vaccine hesitancy (137, 140). (C) Healthcare system-level barriers, such as the failure of obstetricians and midwives to proactively recommend vaccination, a lack of accessible vaccination services, and ineffective information dissemination, are also significant contributors to low vaccination rates (138, 140). Therefore, a comprehensive understanding of the complex determinants that influence vaccine hesitancy is a prerequisite for designing effective interventions.

Currently, interventions for vaccine hesitancy during pregnancy are as follows: (A) It is essential to strengthen education and communication interventions for pregnant women, aiming to improve their awareness of the risks associated with vaccines and diseases, and to correct misconceptions. For example, an Italian study demonstrated that integrating prenatal vaccination education into delivery courses significantly increased influenza vaccination rates among pregnant women (141). (B) Strengthen training and support for healthcare professionals to enable them to proactively recommend vaccination during prenatal care and present scientific evidence to facilitate informed decision-making among pregnant women. Multiple studies have consistently shown that recommendations from healthcare professionals constitute the most critical factor in enhancing influenza vaccine uptake among pregnant women. Strong recommendations from healthcare providers have been shown to increase influenza vaccination uptake among pregnant women by more than tenfold (124, 138, 140, 142). In Greece, physicians’ recommendations were associated with an 18.86-fold increase in the odds of vaccination among pregnant women (138). In Melbourne, Australia, the odds ratio of this association was reported to be 29.7 (140). However, not all healthcare providers are willing to proactively recommend vaccination. Some healthcare providers have insufficient knowledge of vaccines, have doubts about the safety of vaccination during pregnancy, or face limited communication time, all of which can weaken the willingness of healthcare providers to recommend vaccines (143). Therefore, strengthening vaccine-related training for medical staff for ensuring accurate communication of vaccine safety and efficacy information is crucial for improving the vaccination coverage of pregnant women. (C) Implement policy-level interventions, such as incorporating vaccination into routine prenatal care, provide convenient vaccination services (such as administration at the same location and during the same visit), and foster a supportive vaccination environment through public health promotion and policy support. A multicenter survey in Italy revealed that 69.4% of pregnant women preferred to receive vaccinations at their prenatal care institution (144). In Honduras, the influenza vaccination rate reaches 78.1%, with “easy accessibility” cited as a primary driver of vaccine uptake (145). This “one-stop” service model mitigates barriers related to time, transportation, and convenience, making vaccination a natural extension of prenatal check-ups. Additionally, vaccination rates can be effectively improved by designing differentiated health communication strategies for different cultural and social groups, and addressing the concerns and information needs of specific groups. In summary, to improve the coverage of influenza vaccines for pregnant women, multilevel interventions must be implemented to effectively mitigate vaccine hesitancy and realize the public health benefits of immunization planning during pregnancy.

### 5.2 Antiviral treatment strategies and their limitations

#### 5.2.1 Current status of antiviral drug treatments

Antiviral therapy is an important means of relieving symptoms and reducing the duration of influenza infection. As a neuraminidase inhibitor, oseltamivir can effectively inhibit influenza virus replication, alleviate influenza symptoms, and shorten the course of the disease. Therefore, it is widely recommended for early intervention in pregnant women infected with influenza (146). Existing clinical and epidemiological studies have shown that early use of antiviral drugs has significant benefits in reducing the risk of severe illness and related complications in pregnant women. A large-scale study based on the National Health Data System in France showed that the frequency of using antiviral drugs by pregnant women increased yearly from 2010 to 2019, especially for the use of influenza viruses, which reflects the clinical recognition of the safety and effectiveness of antiviral treatment during pregnancy (147).

Clinical reports show that pregnant women with severe influenza can effectively control their condition and ensure maternal and infant safety through comprehensive interventions such as antiviral therapy combined with mechanical ventilation and immunotherapy (7). Additionally, low-dose aspirin treatment has also shown the potential to improve vascular dysfunction related to influenza infection during pregnancy and reduce fetal damage, providing new ideas for clinical treatment (148). In short, the timing and rationale of antiviral treatments are crucial for reducing adverse maternal and infant outcomes related to influenza during pregnancy.

#### 5.2.2 Challenge of antiviral therapy

Although antiviral drugs have obvious advantages, their application still faces some challenges and shortcomings. Firstly, the safety and efficacy data of antiviral drugs during pregnancy are relatively insufficient, and clinicians need to be extremely cautious. Medication during pregnancy should not only consider the therapeutic effect on the mother but also assess the potential risks to the fetus. Presently, commonly used antiviral drugs such as oseltamivir have some application in pregnant women, but limited large-scale and rigorous randomized controlled trial data has led to clinical limitations (149). Secondly, some pregnant women have a low acceptance of antiviral drugs, which may be due to concerns about drug safety and a lack of relevant knowledge, leading to delayed or insufficient medication (146). Thirdly, due to changes in immune and metabolic status during pregnancy, the pharmacokinetic properties of drugs may differ from those taken by nonpregnant women. The pharmacokinetics and metabolic characteristics of existing drugs during pregnancy are not fully understood, which limits the implementation of dose optimization and personalized treatment. Furthermore, antiviral drugs mainly target the replication stage of the virus, and there remains a lack of targeted treatment plans for virus-induced immune inflammatory reactions and tissue damage repair. Therefore, future research should focus on the combined use of antiviral drugs and immune modulators to comprehensively enhance the therapeutic effect of influenza during pregnancy.

## 6 Conclusions and recommendations

Influenza virus, as a highly contagious respiratory pathogen, has multiple subtypes and variants, and its basic characteristics pose a sustained and severe threat to global public health (13). The special immune status and complex maternal fetal barrier function during pregnancy provide theoretical support for susceptibility to influenza virus infection. Although there are certain differences in the exploration of immune mechanisms among different studies, the overall trend tends to emphasize the complexity of immune regulation and its profound impact on infection outcomes, which highlights the direction for the development of future immune intervention strategies.

As an important measure to prevent influenza during pregnancy, vaccination has been validated for its safety and effectiveness in numerous clinical studies. Getting vaccinated against influenza not only significantly reduces the risk of infection in pregnant women, but also protects newborns through maternal antibody transmission, reflecting the core value of vaccines in protecting maternal and infant health. Conversely, nonpharmacological interventions such as personal protection and environmental control have auxiliary effects, but their effectiveness is limited and cannot completely replace vaccination. Therefore, vaccination strategies should be prioritized in clinical and public health practice.

Many challenges remain in current research regarding differences in influenza subtypes, infection mechanisms, and clinical management. Future work needs to strengthen in-depth analyses of the characteristics of different subtypes of influenza infection during pregnancy, combine multicenter and large sample epidemiological data, optimize and improve influenza severity prediction models, and achieve early identification and risk stratification. Simultaneously, promoting the development of early diagnosis technology and personalized management plans for pregnant women will provide a solid foundation for precise prevention and treatment, and help minimize the negative impact of influenza during pregnancy.

Pregnancy status also affects the protective mechanism induced by vaccines, such as the disruption of B cell metabolism in pregnant women after influenza infection, which hinders antibody maturation. However, preexisting vaccine-induced T cell immunity can still play a protective role during pregnancy, reducing adverse outcomes for both mother and baby (150). Additionally, different types of influenza vaccines (such as attenuated live vaccines, recombinant hemagglutinin protein vaccines, and split inactivated vaccines) have differences in immunogenicity during pregnancy and in protective effects on newborns, affecting the transfer efficiency and duration of maternal and infant immune antibodies (130). Therefore, optimizing vaccine design and vaccination timing based on the characteristics of pregnant women, as well as reasonably evaluating the risks and benefits of antiviral therapy, are the focus of current research and clinical practice.

Influenza virus infection during pregnancy is an important issue in the field of public health and has attracted much attention due to its dual threat to the health of pregnant women and fetuses. A deep understanding based on epidemiological characteristics can help develop scientific research and reasonable vaccine promotion strategies, improve the coverage of influenza vaccines for pregnant women, and thereby reduce the overall burden of influenza during pregnancy.
